# Transitions: Athanasios Koukopoulos [AθανάσιΟς κΟυκόπΟυλΟς], M.D. (1931–2013)

**DOI:** 10.1186/2194-7511-1-17

**Published:** 2013-10-08

**Authors:** Daniela Reginaldi, Alexia Koukopoulos, Ross J Baldessarini, Gianni L Faedda, Giuseppe Fazzari, Paolo Girardi, Giorgio Kotzalidis, Giovanni Manfredi, Gian Paolo Minnai, Isabella Pacchiarotti, Michele Raja, Gabriele Sani, Gino Serra, Leonardo Tondo, Eduard Vieta, young pupils, friends, and colleagues from the Aretaeus Association and Lucio Bini Mood Disorder Centers

**Affiliations:** Centro Lucio Bini, Via Crescenzio 42, Roma, 00193 Italy; NESMOS department, Sapienza University at Sant’Andrea Hospital, via di Grottatossa 1045-1039, Roma, 00189 Italy; Harvard Medical School, Mailman Research Center, McLean Hospital, 115 Mill Street, Belmont, MA 02478-9106 USA; ’Lucio Bini’ Mood Disorders Center, 245 East 50th Street, Suite 2A, New York, New York 10022 USA; Chief of Psychiatric Unit, Azienda Ospedaliera Spedali Civili di Brescia Piazzale Spedali Civili, 1, Brescia, Italy; NESMOS department, Sapienza University at Sant’Andrea Hospital, via di Grottatossa 1045-1039, 00189 Roma, Italy; Dipartimento di Salute Mentale, Ospedale San Martino, viale Rockefeller, Oristano, 09170 Italy; Bipolar Disorders Unit, IDIBAPS-CIBERSAM, Institute of Neurosciences, Barcelona University, Hospital Clínic, C/Villarroel 170, Barcelona, 08036 Spain; Centro Gaetano Perusini, via Prisciano 26, Roma, Italy; Department of Biomedical Sciences, University of Sassari, Viale San Pietro 43, 07100 Sassari, Italy; Centro Lucio Bini, Via Cavalcanti 28, 09128 Cagliari, Italy

Athanasios Koukopoulos was born in Chaeronea, a village in the district of Boeotia in central Greece, on 23 November 1931 as the son of Konstantinos and Maria Koukopoulos. His father hoped that he would remain at home to manage the land they owned, but Athanasios preferred to study. His energetic and supportive mother fought for his education. At the end of World War II, following years of exposure to violence and privation, his family moved to Athens just before the Greek civil war of 1946 to 1949. In Athens, Koukopoulos continued his education while playing professional basketball with the prestigious Panatinaikos Sports Club of Athens, which later on presented him with the Golden Clover award. In 1951, he moved to Italy despite remaining hard feelings in Greece following the recent Greco-Italian war of 1940 to 1941 and the subsequent Balkan campaign of the Axis powers in World War II.

**Figure 1:**
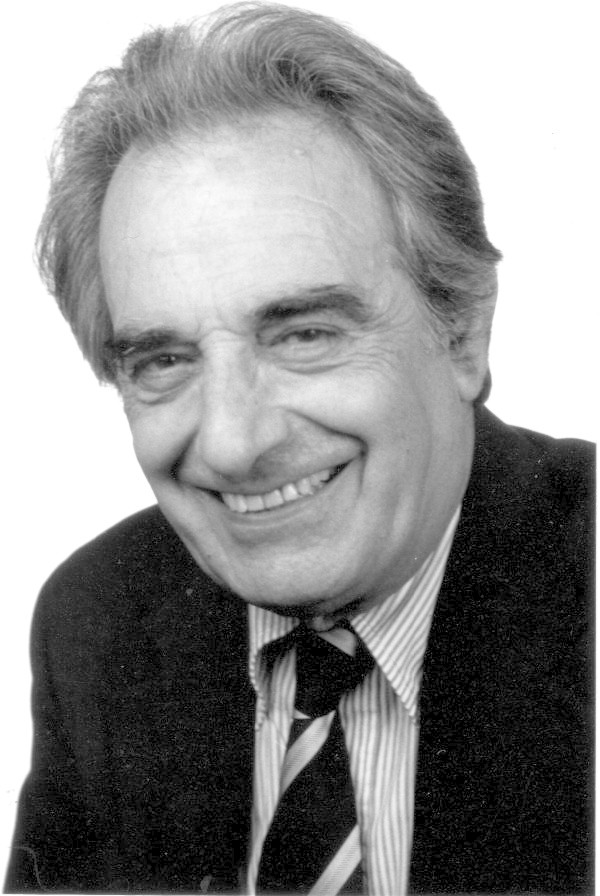
**Athanasios Koukopoulos.**

Koukopoulos settled in Modena where he completed medical school at its university in 1957 while also playing basketball for a regional team (CUS Modena). After serving in the Greek Army as a medical officer, he moved to Rome to complete residency training in psychiatry under the direction of Professor Mario Gozzano. There, he especially admired the courses given by Professor Lucio Bini, one of the developers of electroconvulsive treatment (ECT) in the 1930s, and was impressed by Bini's medical knowledge, intelligence, and teaching skills.

At the age of 32, with Drs. Elena Schiavi and Francesco Montanari, he founded a private psychiatric center, Clinica Belvedere Montello, in Rome in 1963 (closed in 1998). Within a few years, it became one of the most prestigious psychiatric centers in Rome and attracted a prominent international clientele owing in part to Koukopoulos' knowledge of English, French, and German in addition to Greek and Italian. In 1974, he founded the Lucio Bini Center in Rome, in collaboration with other Roman psychiatrists.^a^ Although Koukopoulos lived in Rome throughout his psychiatric career, he never became an Italian citizen, claiming, 'You can never change your parents or place of birth.’

Though not a member of a university faculty, Koukopoulos maintained a keen interest in clinical research and scholarship throughout his career and pursued his interests largely by self-support and based on his personal clinical observations. He developed many intuitions based on his personal observation of many patients over prolonged periods. Many of his principles pertaining to prognosis and treatment response eventually proved to be correct. His method was based on systematic collection and analysis of highly detailed clinical records that captured every clinical encounter and intervening clinical status in extensive case histories - a practice that he maintained to his last days. His most innovative and influential findings pertained to bipolar (manic-depressive) disorder, on which he built an international reputation as a clinical expert. Much of his work has been published in leading psychiatric journals. He also valued young researchers and fostered debate, especially with those with whom he disagreed.

A notable contribution to his interest in bipolar disorder came from an encounter with Dr. Mogens Schou of the University of Aarhus in Denmark in the early 1970s. Following the modern clinical introduction of lithium carbonate in Melbourne, Australia by Dr. John Cade in 1949, Schou, a psychiatrist, had become a major proponent of the use of this inorganic and unpatentable mineral to treat mania and to reduce risk of recurrences of manic-depressive illness. A bipolar disorder patient of Koukopoulos returned from a consultation with Schou and improved dramatically when treated with lithium. This experience led Koukopoulos to visit Schou in Aarhus. They did not always agree but remained friends and colleagues until Schou's death in 2005. Koukopoulos became an early advocate for lithium treatment in Italy, reporting on his clinical findings with the new treatment as early as 1978. These included the first evidence that serum concentrations of lithium were lower during mania and hypomania and higher in depression than in euthymia. These findings supported the hypothesis that the pharmacokinetic clearance of lithium shifted with levels of emotional, behavioral, and physiological arousal and indicated that safe treatment with this potentially toxic agent required corresponding adjustment of doses and close monitoring of blood concentrations (Kukopulos^b^ and Reginaldi 1973;Kukopoulos et al. 1985). In the 1970s, Koukopoulos noted that a strikingly high number of his bipolar disorder patients came from one small village in Sardinia, the third largest Mediterranean island with a current population of 1.5 million. The observation led him to undertake an epidemiological study there, after first consulting with noted psychiatric epidemiologist, Professor Erik Strömgren at the University of Aarhus in Denmark. Progress in this unfunded community survey study was limited by the need to offer free clinical assessments of participants and by lack of cooperation of local physicians who reacted to the project with considerable suspicion. However, the project led to unprecedented interest in seeking psychiatric consultation from all over the island and led to the foundation of a second Lucio Bini Center in Cagliari, founded by Koukopoulos' colleague, Dr. Leonardo Tondo and his colleagues in 1976.^c^

Koukopoulos had insights that have had important influence on the study and treatment of recurrent major mood disorders. At a time when the great majority of severe psychiatric disorders were diagnosed as 'schizophrenia’ in Italy and many other countries, he recognized prominent affective psychopathology in many cases. Importantly, 'major depression’ became the dominant paradigm for mood disorders by the time of introduction of the American Psychiatric Association's Diagnostic and Statistical Manual Third Edition (DSM-III) in 1980. However, Koukopoulos recognized that mania and hypomania were important components in many cases of episodic mood disorders, which should be recognized and treated as bipolar disorder. One of his well-known sayings was that 'Mania is the fire of the disease, depression its ash.’

In the 1980s, Koukopoulos focused on the sequence of mania-like and depressive episodes that characterized the illness course in individual cases of bipolar disorder. He identified some with mania followed by depression and an illness-free interval (MDI pattern), others with depression preceding mania and a stable interval (DMI), and still others with long, continuous cycles with no good interval (CC), as well as the better known pattern of rapid-cycling at rates of four or more recurrences within 1 year. He observed that the beneficial effect of long-term treatment with lithium was greatest in bipolar disorder patients with the MDI recurrence pattern compared to others (Kukopulos et al. 1980), and this finding has been replicated independently several times (Koukopoulos et al. 2013). He and his collaborators also proposed that antidepressants can decrease beneficial responses to prophylactic treatment with lithium (Reginaldi et al. 1981) and sometimes induce mania-like reactions and increase rates of shifting into and out of manic episodes (Kukopulos et al. 1980,1983;2003;Tondo et al. 1981). Such unfavorable responses seem to be particularly likely among patients with a DMI course (Koukopoulos et al. 2013). Koukopoulos and his colleagues were among the first to recognize the limitations and potential risks associated with antidepressant treatment in bipolar depression (Reginaldi et al. 1981).

One of Koukopoulos' most important contributions to research on bipolar disorders was his theory of the 'primacy of mania’ and excited states in the course of mood disorders. The theory arose in 1973 based on findings suggesting that depression could be avoided by suppressing mania in bipolar disorder patients (Koukopoulos et al. 2006). Such observations suggested that this effect may help to account for the long-term prophylactic or mood-stabilizing effects of the antimanic agent lithium (Koukopoulos and Ghaemi 2009).

Koukopoulos also was a scholar of the contributions of nineteenth century French psychiatrists, including Esquirol and Falret, as well as the German psychiatrists Griesinger, Hecker, Kahlbaum, and Kraepelin. Among ideas arising from their work, he reported on the contribution of premorbid affective temperaments to the course of mood disorders and their treatment responses (Koukopoulos 2003). He was also influenced by the classical contributions of Hippocrates and Aretaeus, who described cases that resemble modern bipolar disorder patients. During a clinical meeting, he politely criticized a clinical report made by a younger psychiatrist by reminding him that the power of the clinical analyses of cases described by Hippocrates was due to the fact that he was, before anything else, a poet, and he proposed that only a poet could have such a profound and true understanding of human diseases.

The topic of simultaneous expression of mania-like and depressive symptoms stirred Koukopoulos' interest from the early 1990s (Koukopoulos et al. 1992). This interest followed his study of contributions on the topic by German psychiatrists of the twentieth century who followed the seminal contributions of Wilhelm Weygandt in the 1890 s to the concept of 'mixed-states’ in mood disorders. In keeping with recommendations of nineteenth century predecessors including Falret, Kahlbaum, and Kraepelin, Koukopoulos underscored the importance of longitudinal observation of manic-depressive patients. He was also struck by the clinical significance of agitated or dysphoric depression for risk of suicide (Koukopoulos et al. 1992;Sani et al. 2011). Importantly, he proposed that agitated depressions and other mixed-states of bipolar disorder need to be treated differently from other types of depression (Koukopoulos et al. 1989,1992,2007;Koukopoulos and Koukopoulos 1999;Benazzi et al. 2004). He recently criticized the new fifth edition of the DSM (DSM-5) for its proposed diagnostic criteria for 'mixed depression’ which he considered misleading. An editorial with these views appeared on the internet on the same day as his death in April 2013 Koukopoulos and Sani (2013).

Koukopoulos trained and influenced three generations of Italian and European psychiatrists, despite sometimes being viewed with raised eyebrows from Italian academia, often envious of his international recognition and respect. In 2009, he was given a lifetime achievement award^d^ from the International Review of Bipolar Disorders for his research on bipolar disorders, and in 2012, the Società Italiana di Psicopatologia (Italian Society for Psychopathology) awarded him a prize for excellence in clinical psychiatric research.

His clinical research was largely unfunded, empirical, and based on his observations among the countless patients he personally cared for. Koukopoulos was a strong advocate of educating professionals as well as patients and their families about bipolar disorder. In 1998, he founded Aretaeus, a nonprofit organization, devoted to public and professional education on the disorder, in honor of the Greco-Roman physician from Cappadocia (in modern central Turkey) who described cases that may have represented bipolar disorder in 150 CE.

Deep understanding and compassion for patients suffering from mental illness and their families guided Koukopoulos in his clinical work, research, teaching, and mentoring. He committed his life to relieving suffering of patients with selfless dedication and profound humanity. He frequently devoted extra time to their care, sometimes for hours at a time with no increase in fee. Always available for advice, he never turned off his cell phone: 'A life-saver in moments of crisis,’ he used to say. He made great efforts to reduce feelings of stigma in and about psychiatric patients, kept them and their families well informed about individual illnesses and progress, and worked with patients collaboratively to develop plans for their treatment - all of this in times when the doctor-patient relationship was mostly unidirectional. Sometimes, he invited patients to his home for dinner and would see them even during vacation times when he was with an entourage of family and friends. He also welcomed patients to attend the many international meetings that he organized almost yearly and routinely waived their registration fee.

His close observation of patients' clinical status allowed him to develop general rules about the course of illnesses and treatment responses that are now widely accepted by the scientific and clinical communities. His clinical discernment and immense experience with thousands of patients suffering from manic-depressive illness prompted him to emphasize the crucial importance of ECT as well as lithium and other modern medicinal treatments Kukopulos et al. (1977). In his support of the value of ECT, he paid tribute to his maestro, Lucio Bini, after whom he named the mood disorder centers in Rome, Cagliari, and a third in New York City that was founded by Drs. Gianni L. Faedda and Paolo Decina in 1991. He courageously opposed those who wished to abolish ECT without considering available clinical and research findings and inspired the foundation of the Italian Association for Electroconvulsive Therapy (AITEC) in 2010.

There was something deeper and more mysterious that animated the life and profession of Athanasios Koukopoulos - something hard to define. He was a professed atheist, but he had a spiritual and ethical view of life and regarded some principles and feelings as sacred, particularly love and friendship. This perspective helped him explore the most secret aspects of the human soul. Those who met him are proud to have known this apparently calm, moderate, and generous man to be always polite but at times combative and full of inner fire. His political thinking also was moderate. He was a prominent member of the Panhellenic Liberation Movement (PAK) and actively opposed the Greek dictatorship in 1967 to 1974. For years, he could not return to Greece, where he would have been arrested, but he sometimes hosted representatives of PAK at his home in Rome.

Koukopoulos died in the early morning of 20 April 2013 of cancer at his apartment in Rome, surrounded by his long-time partner, Daniela Reginaldi, their two daughters, Alexia and Arianna, his son, Harilaos, and his four beloved grandchildren. Following a brightly burning career, his ashes rest in the non-Catholic or Protestant cemetery in Rome.

## Endnotes

^a^Drs. Antonio Bernabei, Benedetto Caliari, Adele De Pascale, Paolo Girardi, Daniela Reginaldi, Rosanna Izzo, and Leonardo Tondo. The clinic was previously Clinic of Psychology and Psychiatry whose co-founders in 1970 were Drs. Andrea Dotti, Alberto Gaston, Nicola Lalli, and Paolo Pancheri.

^b^In 1985, *Koukopoulos* legally changed his family name from *Kukopulos* to retain its original Greek spelling.

^c^In collaboration with Drs. Maria Cantone, Gianfranco Floris, Paolo Laddomada, Gian Paolo Minnai, Gino Serra, Francesco Toccafondi.

^d^Previous recipients: Hagop Akiskal, Jules Angst, and Frederick Goodwin.
